# Modulation of the Inflammatory Process through the Control of Cyclooxygenase Using Peptides Obtained from Lithobates Catesbeianus Identified by Molecular Docking

**DOI:** 10.1021/acsomega.5c02159

**Published:** 2025-05-14

**Authors:** Patricia da Silva Mattosinhos, Silvania Mol Pelinsari, Raul Santos Alves, Manoela Maciel dos Santos Dias, Rômulo Dias Novaes, Edvaldo Barros, Marcos Rogério Tótola, Tiago de Oliveira Mendes, Reggiani Vilela Gonçalves

**Affiliations:** † Department of General Biology, Federal University of Viçosa, Viçosa 36570-000, MG, Brazil; ‡ Department of Animal Biology, Federal University of Viçosa, Viçosa 36570-000, MG, Brazil; § Department of Structural Biology, Federal University of Alfenas, Alfenas 37132-440, MG, Brazil; ∥ Biomolecule Analysis Center (NUBIOMOL), Federal University of Viçosa, Viçosa 36570-000, MG, Brazil; ⊥ Departament of Microbiology, Federal University of Viçosa, Viçosa 36570-000, MG, Brazil; # Department of Biochemistry and Molecular Biology, Federal University of Viçosa, Viçosa 36570-000, MG, Brazil

## Abstract

Inflammatory diseases encompass a wide range of disorders that affect different systems of the body, such as the skin, joints, and gastrointestinal tract. Notable examples of such disorders include psoriasis, rheumatoid arthritis, and Crohn’s disease. Research highlights the therapeutic potential of animal-derived peptides under these conditions. Advances in discovering promising drugs involve advanced bioinformatics techniques, such as molecular docking combined with *in vitro* studies, which have shown consistent results. This study aimed to explore and characterize peptides derived from the enzymatic hydrolysis of bullfrog skin proteins with the potential to modulate multiple inflammatory pathways, particularly through cyclooxygenase (COX) inhibition and IL-6 downregulation. Bullfrog skin was lyophilized, and peptides were extracted through enzymatic hydrolysis. Peptide fractionation was performed using solid-phase extraction, and mass spectrometry was performed on the target fraction. A molecular docking analysis was subsequently utilized to predict the interactions of the peptides with the COX active site. The peptide sequences were investigated for their potential to inhibit the COX enzymes through a colorimetric inhibition assay. The IL-6 expression was evaluated on the selected peptide sequence using a Murine Mini ABTS IL-6 enzyme-linked immunosorbent assay (ELISA) development kit. Based on the results, the bullfrog skin hydrolysates, especially those obtained through trypsin digestion, exhibited a significant dose-dependent enhancement in the cell viability of RAW 264.7 macrophages. Notably, the *F*4 fraction, isolated by solid-phase extraction, demonstrated the most pronounced effect. Mass spectrometry analysis of the *F*4 fraction identified 71 low-molecular-weight peptide sequences corresponding to different bullfrog proteins. Four peptides (P1, P2, P3, and P4) were selected for synthesis based on molecular docking results, which predicted a high binding affinity and favorable interactions with the COX active site, especially for peptide GPSGPAGARGDK (P3). Despite the strong binding affinity of P3, biological proof-of-concept studies revealed that SGHPGAMGPVGPR (P1) exhibited the most significant results, effectively inhibiting total COX activity and downregulating IL-6 expression in RAW 264.7 macrophages at a concentration of 1 mM. The P1 peptide exhibited structural stability and demonstrated a superior ability to modulate the inflammatory response. Although some discrepancies between molecular docking and *in vitro* results were observed, this study highlights the importance of integrative analyses in enhancing success rates for identifying viable therapeutic candidates. Overall, the findings indicate that peptide P1, identified within the *F*4 fraction, is a promising candidate for further optimization as a cyclooxygenase inhibitor, with potential applications in the development of biopharmaceuticals for the treatment of inflammatory skin diseases. Further controlled studies are necessary to elucidate additional mechanisms underlying its anti-inflammatory properties and to refine its therapeutic potential.

## Graphical abstract

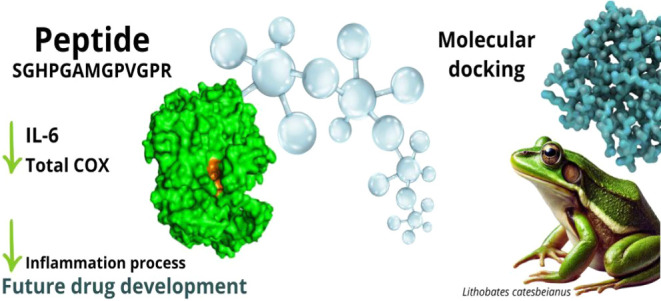

## 1 Introduction

Inflammatory diseases encompass a broad spectrum of disorders, including rheumatoid arthritis, inflammatory bowel disease, and psoriasis, characterized by pain, swelling, erythema, and impaired tissue function.^1^ These conditions disrupt the metabolic balance and structural integrity of affected tissues, compromising their adaptive responses to physical, chemical, and microbiological stimuli.^2^ Inflammatory chronic diseases significantly affect patients’ quality of life, with their impact further exacerbated by the limited effictiveness of current therapeutic options.^3^ The pathogenesis of inflammatory diseases remains incompletely understood due to the involvement of diverse immunological mediators, growth factors, genetic factors, and signaling pathways driving the associated molecular and morphological abnormalities. In recent years, therapeutic research has increasingly focused on developing bioactive compounds with anti-inflammatory properties.^4^ Notably, animal-derived peptides have emerged as promising candidates in treating inflammatory conditions, as highlighted by a previous report,^5^ offering new avenues for innovative and targeted interventions.

Peptides derived from natural sources exhibit pharmacological, antioxidant, and antimicrobial properties. Furthermore, they can be released under enzymatic hydrolysis conditions, demonstrating great abundance, high digestibility, nutritional relevance, a low production cost, and limited toxicity.^6^ Despite the limited data on the technological applicability and biological effects of animal-derived peptides in skin diseases, *in vitro* studies and animal models have demonstrated their significant anti-inflammatory and antioxidant activities.^5^ Thus, mechanisms such as the inhibition of proinflammatory processes mediated by nuclear factor kappa B (NF-κβ) and the activation of the Nrf-2 antioxidant pathway have been particularly associated with the cutaneous therapeutic effects of animal-derived peptides.^7–9^

Evidence shows that animal peptides derived from bullfrog skin can be a relevant alternative for inflammatory disease treatment.^10,11^ Considering the need to advance the discovery of effective drugs for inflammatory skin disease treatment, the integration of advanced bioinformatics techniques, such as molecular docking coupled with *in vitro* studies, has been identified as a promising approach. These methodologies facilitate the rational biotechnological development of more effective therapeutic agents. Thus, the identification of a strong drug-target affinity from computational screening techniques has potential relevance in the search for favorable anti-inflammatory and antioxidant biological responses.^12^ Molecular docking is used to analyze the interaction between proteins and ligands, indicating the binding mode and affinity strength in the interactions between ligands and molecules with a known stable structure.^13^ Integrating *in vitro* studies as a validation step for docking analyses is a crucial part of biopharmaceutical discovery, enabling investigations into different aspects such as absorption, target regulation, metabolic stability, and toxicity.^12^ This approach has been extensively employed as a valuable strategy in drug discovery research, explicitly targeting the identification of novel bioactive molecules including bioactive peptides. To understand the role of this molecule inside cells, we have some essential biological targets that we can highlight, such as cyclooxygenases, which are enzymes involved in the inflammatory control process.

The biosynthesis of prostaglandins from arachidonic acid is catalyzed by cyclooxygenase (COX), which exists as COX-1 and COX-2. Arachidonic acid is, in turn, released from the cell membrane by neopathological stimuli.^14^ COX inhibitors interfere with this catalytic and disease-initiating process.^15,16^ There is evidence that the simultaneous inhibition of COX-1 and COX-2 may contribute to the prevention and treatment of certain types of inflammatory chronic diseases, including diabetes, skin diseases, and cancer, by interfering with pathways associated with the control of proinflammatory cytokines, cell proliferation, and angiogenesis.^17^ In specific clinical contexts, this inhibitory duality could also be explored in combination therapies, enhancing therapeutic effects in protocols to treat chronic pain or refractory inflammations. In this regard, the discovery of selective COX-1 and COX-2 inhibitory peptides has been proposed as a strategy for developing next-generation anti-inflammatory drugs, aiming to create more potent and safer inhibitors.^18^ This study integrated *in vitro* and *in vivo* approaches to explore and characterize COX-1 and COX-2 inhibitory peptides derived from the enzymatic hydrolysis of bullfrog skin peptides. We investigated the molecular interactions between the peptides and the active sites of COX-1 and COX-2, providing insights into their potential as selective or dual inhibitors and their capacity to modulate the inflammatory process.

## 2 Experimental Section

### 2.1 Sample Collection and Animal Care

This experiment used 10 bullfrog specimens (males, 250 ± 15 g, and 38 weeks old) obtained from the Frog Experimental Farm of the Universidade Federal de Viçosa (UFV). The animals were subjected to controlled environmental conditions for adaptation over 3 days (27 ± 1 °C temperature and 12/12 h light/dark cycles). Frog feeding was based on commercial extruded feed containing 44 g of crude protein and 1% Musca domestica (Linnaeus, 1758) larvae as the bait, administered twice daily. The animals were weighed, anesthetized with 0.1% benzocaine, and euthanized by cranial concussion according to the recommendations of the American Veterinary Medical Association (AVMA, 2001). Immediately after euthanasia, the skins were separated, washed in a sterile saline solution (0.9% NaCl, previously cooled at 4 °C), and stored at −80 °C for subsequent analyses. All experiments involving animals were approved by the Animal Research Ethics Committee of the Universidade Federal de Viçosa (protocol 824/2018).

### 2.2 Preparation of Bullfrog Skin Hydrolysates

Bullfrog skin peptides were extracted by enzymatic hydrolysis using four independent enzymes: alcalase, pepsin, papain, and trypsin. Briefly, the substrate (bullfrog skin) and enzymes were mixed in a 1/100 ratio (w/w). Each mixture was incubated for 8 h, considering the optimal temperature for each enzyme, with constant stirring. After incubation, the mixtures were subjected to 10 min of heat treatment at 80−95 °C in a water bath for enzyme inactivation. The resulting hydrolysates were lyophilized and then tested at different concentrations (25, 50, 100, and 200 μg/mL) for viability in RAW 264.7 macrophage cells. The hydrolysate showing the highest viability was chosen for subsequent fractionation.

### 2.3 Solid-Phase Extraction

The peptide fractionation was carried out by solid-phase extraction. Initially, 10 mg of the lyophilized extract was dissolved in 1 mL of distilled water and centrifuged at 10,500*g* for 10 min. The supernatant was collected by using a Whatman ODS-5 solid-phase extraction column. Elution was performed using a 0 to 85% acetonitrile gradient in 0.1% trifluoroacetic acid, resulting in 10 fractions (*F*1 = 0%, *F*2 = 5%, *F*3 = 15%, *F*4 = 25%, *F*5 = 35%, *F*6 = 45%, *F*7 = 55%, *F*8 = 65%, *F*9 = 75%, and *F*10 = 85%). For the bioactivity assay, 2 mL of each fraction was lyophilized and resuspended in 1 mL of the culture medium, and 200 μL of this solution was tested for viability in RAW 264.7 macrophages. The fraction exhibiting the highest viability was selected for further testing at different concentrations (3.125, 6.25, 12.5, and 25 μg/mL) on RAW 264.7 macrophages. A portion of this fraction was dried by using a vacuum centrifuge and stored at −20 °C until mass spectrometry analysis.

### 2.4 Cell Viability Assay

After identifying the fractions, they were tested for cell viability assays conducted on RAW 264.7 macrophages and NIH/3T3 fibroblasts using the 3-(4,5-dimethylthiazol-2-yl)-2,5-diphenyltetrazolium bromide (MTT) reduction method.^19^ Cells were cultured in Dulbecco’s modified Eagle’s medium (DMEM) supplemented with 10% fetal bovine serum (FBS) and 100 U/mL penicillin/streptomycin. The absorbance was then measured using a microplate reader (Multiskan FC, Thermo Labsystems, Franklin, MA) set at 570 nm.

### 2.5 Mass Spectrometry

The target fraction was dissolved in 20 μL of 0.1% (v/v) formic acid solution, with the addition of 2% (v/v) liquid chromatography mass spectrometry (LC-MS)-grade acetonitrile. The sample was placed in appropriate tubes for analysis using the nano LC-MS/MS system. After preparation, 1 μL of the solution was injected into a nanoAcquity ultra-performance liquid chromatograph (UPLC) (Waters). This system included a capture column (nanoAcquity UPLC 2GV/MTrap 5 μm Symmetry C18, 180 μm × 20 mm), operating at a flow rate of 7 μL/min for 3 min. Peptides were separated using a nanoAcquity UPLC 1.8 μm HSS T3 column (75 μm × 200 mm) at a flow rate of 0.2 μL/min. The mobile phase consisted of water acidified with 0.1% formic acid (solvent A) and acetonitrile acidified with 0.1% formic acid (solvent B). The chromatographic separation protocol was as follows: 2% B for 1 min; a gradient from 2 to 30% B over 209 min; a gradient from 30 to 85% B for 10 min; a hold at 85% B for 5 min; a gradient from 85 to 2% B for 5 min; and a hold at 2% B for 10 min, totaling 240 min of analysis. The eluted peptides were automatically injected into a MAXIS 3G mass spectrometer (Bruker Daltonics, Germany) utilizing a CaptiveSpray ionization source. Peptide analysis followed the specific proteomic method (IE_captive_nov2019), with a drying gas flow of 3 L/min, an ionization source temperature of 150 °C, and a transmission voltage of 2 kV. Raw data were converted into a mass list in the *mzXML format using CompassXport version 3.0 software (Bruker Daltonics, Germany) and analyzed using PEAKS software.

### 2.6 Data Analysis from Mass Spectrometry

The mass lists in the *mzXML format were compared with the protein sequence database of the Lithobates catesbeianus species (downloaded on 31/01/2022, containing 29.178 entries) maintained by the UniProt Consortium. The comparison was performed using PEAKS software version 7.0 (Bioinformatics Solutions Inc., Canada). The parameters adopted in this study included enzymatic digestion by trypsin, allowing for up to two missed cleavages: carbamidation of cysteine as a fixed modification and oxidation of methionine as a variable modification. The error tolerance for the parent ion was 20 ppm; for fragments, it was 0.6 Da, considering the analysis of ions with charges of +2, + 3, and +4. Protein identification was established when at least two unique peptides were identified with a false discovery rate (FDR) of less than 1%. Proteins categorized as “uncharacterized” were analyzed using BLAST software version 2.4.0.^20^ In this analysis, it was possible to determine which proteins deposited in the nonredundant protein database (nr) of the National Center for Biotechnology Information (NCBI) showed greater similarity to the sequences of proteins classified as “uncharacterized.” The estimation of the relative quantity of each peptide was determined by the ratio between the MS1 peak area corresponding to each peptide and the sum of the peak areas of all identified peptides with the sum of all peak areas considered as 100%. The MS/MS peptide spectra are available in the Supporting Information (Appendix 1).

### 2.7 Molecular Docking

Molecular docking was performed using AutoDockVina 1.1.257 software, following a previously reported methodology with adaptations.^21^ The receptors used were human COX-1 and COX-2, obtained from the Protein Data Bank (PDB ID: 6Y3C), with a resolution of 3.36 Å (Miciaccia et al., 2020) and 2.34 Å (PDB ID: 5IKR) (Orlando and Malkowski, 2016), respectively. AutoDockTools was employed for receptor editing, including the removal of water molecules, the addition of nonpolar hydrogen atoms, and the calculation of protein charges. The file was then converted to the PDBQT format (Protein Data Bank, partial charge [Q] and atom type [T]). The ligands and peptides developed in this study were drawn using Marvin Sketch 17.28.049 software, with all hydrogen atoms explicitly displayed. The files were saved in 3D in the PDB format. Subsequently, PyRx Python Prescription 0.861 software was used to convert the files to the PDBQT format. This conversion is essential, as it incorporates partial charges and atom types, critical for accurately simulating the electrostatic interactions and binding affinities during the molecular docking process. Potential inhibitors were docked into the enzyme using AutoDockVina 1.1.257 software. A rectangular prism with dimensions 76 × 60 × 63 Å (*x*, *y*, and *z* axes, respectively) was created to allow the ligands to interact throughout the protein using a nondirected docking strategy. The prism center was positioned at *x* = 32.0874, *y* = 6.7681, and *z* = 58.8835 Å. The inhibition constant (*K*_i_) was calculated from the binding free energy (Δ*G*) using the following equation: Δ*G* = *RT* ln (*K*_i_), where *R* is the universal gas constant (1.987 cal/mol·K), and *T* is the temperature in Kelvin (298 K). More negative Δ*G* values indicate a stronger binding affinity and, consequently, a greater inhibition of the COX-2 active site.

## 3 Biological Approach

### 3.1 Peptide Synthesis

For the biological tests (cell viability analyses, COX inhibition assay, and IL-6 immunoassay), the 4 peptides that presented the best binding affinity energy by molecular docking were chosen and synthesized. The peptides were synthesized with 90% purity and at a scale of 10 mg by AminoTech Industry and Commerce (Sao Paulo, SP, Brazil) (for more information, see the Supporting Information (Appendix 2)).

### 3.2 COX Inhibition Assay

The *in vitro* total cyclooxygenase inhibition was evaluated using a chemiluminescent enzymatic kit (Cayman Chemical, MI), according to the manufacturer’s protocol. The RAW 264.7 cell line was purchased from the American Type Culture Collection (Rockville, MD) and routinely grown in DMEM supplemented with 10% fetal bovine serum (FBS). The cells were subcultured every 3 to 4 days at 1 × 10^8^ cells/mL. The positive control group was exposed to lipopolysaccharide (LPS) at 1 μg/mL for 4 h to induce inflammation. Cells were collected and centrifuged at 600*g* for 10 min at 4 °C. The supernatant was carefully removed and cells were resuspended in 2 mL of DMEM, resulting in 1 × 10^8^ cells/mL.

### 3.3 IL-6 Immunoassay

Quantification of IL-6 was determined using the Murine Mini ABTS IL-6 ELISA kit (PeproTech, Cat No. 900-M50), according to the manufacturer’s instructions. The absorbance was measured at 405 nm, and IL-6 concentrations were quantified based on the IL-6 standard curve (0−4000 pg/mL).

### 3.4 Cell Viability Assay

A second cell viability assay was conducted to specifically evaluate the four most promising peptides (P1, P2, P3, and P4) at a concentration of 1 mM, selected based on their superior performance in the molecular docking analysis. RAW 264.7 macrophage cells were cultured in Dulbecco’s modified Eagle’s medium (DMEM) supplemented with 10% fetal bovine serum (FBS) and 100 U/mL penicillin/streptomycin. Cells were treated with the different peptides (1 mM) for 24 h, and cell viability was assessed using the 3-(4,5-dimethylthiazol-2-yl)-2,5-diphenyltetrazolium bromide (MTT) reduction method.^19^ The absorbance was measured at 570 nm using a microplate reader (Multiskan FC, Thermo Labsystems, Franklin, MA). Notably, this analysis differs from the initial viability test performed on peptide fractions as it was specifically designed to evaluate the safety profile of the selected individual peptides for subsequent *in vitro* assays.

## 4 Statistical Analysis

Statistical analysis was conducted using GraphPad Prism 7 software (GraphPad Software Inc., San Diego, CA). Data are presented as mean ± standard deviation (mean ± SD). The Shapiro−Wilk test was applied to assess the normality of data distribution. For data exhibiting confirmed normality, a one-way analysis of variance (ANOVA) was performed, followed by Tukey’s post hoc test for pairwise comparisons. The statistical significance was determined at *p* < 0.05.

## 5 Results and Discussion

The hydrolysates extracted from bullfrog skin using four distinct enzymes positively impacted RAW 264.7 macrophage viability at varying concentrations (Figure 1a). Papain and trypsin hydrolysate demonstrated the most significant effect on cell viability among the four tested hydrolysates (122.7 ± 4.04% cell viability at 25, 50, and 200 μg/mL). Trypsin was chosen for further separation through solid-phase extractions because it has advantages over papain regarding peptide hydrolysis due to its high specificity, resulting in predictable fragments useful in studies such as mass spectrometry.^22,23^ Additionally, trypsin operates under conditions close to physiological pH (7.5−8.5), preventing excessive protein degradation and ensuring greater control in producing bioactive peptides for pharmaceutical and industrial applications.^24,25^ Trypsin is ideal for applications that require precision, as it is widely purified and consistent. Conversely, papain operates less specifically, acting on a broad range of peptide bonds, which can result in less controlled degradation products. Furthermore, its instability under cold storage conditions, acidic pH, and the presence of ethanol, combined with its tendency for non-native aggregation, compromises its efficiency and experimental reproducibility. These factors make trypsin a more reliable choice for experiments requiring greater control over protein digestion and enzymatic stability.^26,27^ After using trypsin extraction, we obtained 10 peptide fractions (*F*1 until *F*10), and three fractions (*F*2, *F*9, and *F*10) demonstrated cytotoxic effects, while five fractions (*F*1, *F*3, *F*5, *F*7, and *F*8) showed no impact on cell viability. Additionally, the two fractions (*F*4 and *F*6) increased cell viability (Figure 1b). The most positive results for cell viability were observed following exposure to the *F*4 fraction (140 ± 3.51% cell viability). Therefore, the *F*4 fraction was chosen, isolated, and subsequently assessed at different concentrations to determine its effect on cell viability. A significant increase in cell viability was observed in both RAW 264.7 macrophages (178 ± 13.45% cell viability at 25 μg/mL; Figure 1c) and NIH/3T3 fibroblasts (198 ± 11.53% cell viability at 25 μg/mL; Figure 1d). Our results showed a dose-dependent effect, with the best results at 25 μg/mL in RAW 264.7 macrophages. The *F*4 fraction was then selected for mass spectrometry analysis to identify peptides responsible for increased cell viability. We believe that these results can be attributed to the presence of bioactive compounds that promote cell viability and integrity. Among the compounds that may be present in the *F*4 fraction, collagen peptides stand out, as they stimulate extracellular matrix production and promote cell viability and integrity. Previous studies indicate that collagen-derived peptides have multiple biological activities, including antioxidant, anti-inflammatory, and anticancer effects.^28–30^ Other compounds in the *F*4 fraction that may be present are hematopoietin and vimentin proteins. Hematopoietin, involved in cell differentiation, can influence immune cell proliferation, including macrophages.^31,32^ Vimentin, an intermediate filament protein, may also play a role in cell migration, especially in fibroblasts.^33,34^

**Figure 1:**
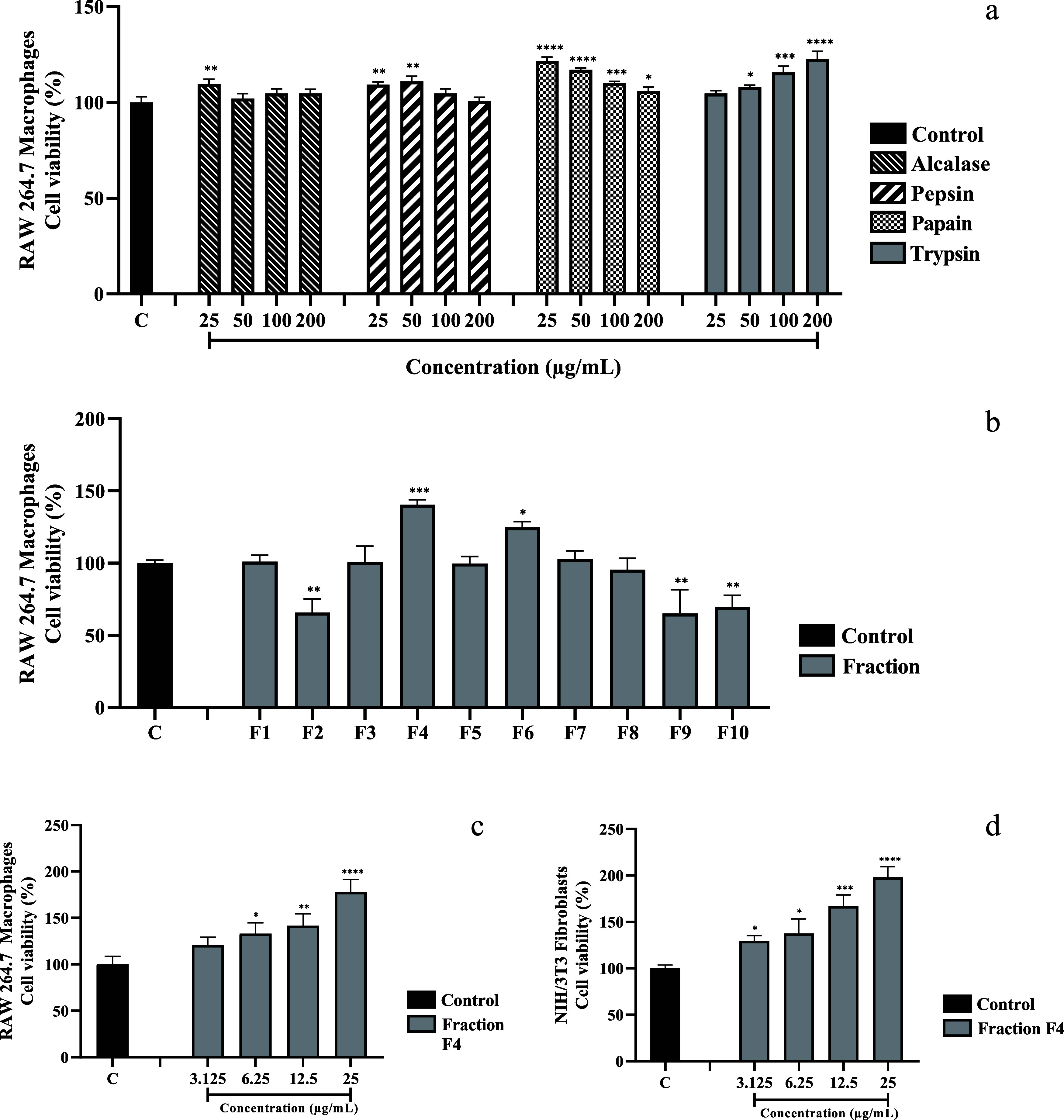
Effect of peptide fractions on the cell viability. RAW 264.7 macrophages were treated separately with bullfrog skin hydrolysates using four different enzymes separately (alcalase, pepsin, papain, and trypsin) at different concentrations (25, 50, 100, and 200 μg/mL) for 24 h (a). RAW 264.7 macrophages were treated separately with ten fractions obtained from the trypsin-derived hydrolysate, in which 2 mL of each fraction was lyophilized, resuspended in 1 mL of the culture medium, and applied to the cells for 24 h (b). RAW 264.7 macrophages were treated with the *F*4 fraction at different concentrations (3.125, 6.25, 12.5, and 25 μg/mL) for 24 h (c). NIH/3T3 fibroblasts were treated with the *F*4 fraction at different concentrations (3.125, 6.25, 12.5, and 25 μg/mL) for 24 h (d). The data (*n* = 3) are expressed as mean and standard deviation. Statistical difference *vs* control group *p < 0.05, **p < 0.01, ***p < 0.001, and ****p < 0.0001 (one-way ANOVA with Tukey’s post hoc test).

Following the promising results related to the viability of RAW 264.7 macrophage cells after *F*4 fraction exposure, our objective was to identify the specific peptides within the *F*4 fraction responsible for these effects. Mass spectrometry exclusively revealed low-molecular-weight peptides (<5 kDa) within the *F*4 fraction. Seventy-one peptide sequences were identified, corresponding to bullfrog proteins such as collagen α-1 (I) chain, collagen α-1 (XII) chain isoform X3, collagen α-2 (I) chain, collagen α-3 (VI) chain isoform X6, collagen α-3 (VI) chain isoform X11, hemoglobin subunit α, hemoglobin subunit β, monomeric α-macroglobulin, albumin, vimentin-4, annexin, tropomyosin-1 α, fibrinogen C-terminal, actin cytoplasmatic 2, AhpC-TSA, 2-phospho-d-glycerate hydrolyase, histone H4, and intermediate filament rod domain (Table 1). Peptides from bullfrog skin are often rich in hydrophobic and charged amino acid residues, which enhance their interactions with cell membranes and contribute to their bioactivity. Compared to antimicrobial peptides from insects or other amphibians, bullfrog-derived peptides demonstrate higher stability and a broader spectrum of biological functions,^5^ likely due to their structural diversity and unique sequence motifs. It is already known that all of the proteins described above have an important role in maintaining the structural integrity of tissues.^37^ For example, the α-1 (I), α-1 (XII), α-2 (I), and α-3 (VI) collagen chains stand out for their importance in the organization of connective tissue.^38^ In addition to structural proteins, plasma proteins, such as albumin and hemoglobin, play essential roles in the body. Albumin is involved in the transport of various substances and has been explored in the development of drug carriers due to its biocompatibility and ability to bind to different molecules.^39^ On the other hand, hemoglobin, composed of α and β subunits, plays a fundamental role in oxygen transport in the blood. Peptides derived from this protein have been investigated for their antioxidant and antimicrobial properties, suggesting innovative therapeutic applications for combating infections and neutralizing free radicals.^40^ Other proteins in the cytoskeleton, such as vimentin, cytoplasmic actin 2, and tropomyosin-1 α, are fundamental in modulating the immune system and inflammatory response, highlighting their relevance for the development of new therapeutic approaches for autoimmune and degenerative muscle diseases.^41^ Annexin and fibrinogen also play a significant role in the biomedical context. Annexin is involved in inflammatory and coagulation processes, and its derived peptides may hold promise for the treatment of cardiovascular and inflammatory diseases. Meanwhile, peptides derived from fibrinogen have been studied for their hemostatic properties, with potential applications in developing surgical sealants and agents for bleeding control.^42–44^ Thus, the characterization and utilization of peptides derived from these proteins introduce new possibilities for the development of innovative therapeutic agents. Whether in tissue regeneration, inflammatory process regulation, immune system modulation, or the design of new drug delivery systems, these peptides represent a vast and valuable field of biomedical applications that deserve extensive investigation.

**Table 1:** Protein-Related Peptide Sequences Found in the Hydrolyzed Extract of Bullfrog Skin by Trypsin^a^

protein accession (18)	protein description	peptides (71)	mass (Da)	relative abundance (%)
A0A2G9S0U7	collagen α-2 (I) chain	SGNRGEGGPSGPAGITGPSGPRGPAGPQGVR	2783,3811	2,13
		SGHPGAMGPVGPR	1218,5928	1,00
		GPSGPSGPPGKEGR	1278,6316	1,29
		GAPGERGEAGPAGPTGFAGPPGAAGHTGAKGDR	2927,4021	0,32
		IGPAGSAGSR	871,4512	0,92
		AGGIGPAGSR	841,4406	0,94
		GIPGPAGPAGPSGAR	1260,6575	0,00
		GPAGAAGAPGPAGGPGDRGESGPAGPSGVAGPR	2708,3013	0,00
		SLNQQIEVILTPEGSRK	1911,0425	11,18
A0A2G9SH44	collagen α-1 (I) chain	GLTGPIGPPGPGGAPGDKGEAGPAGPAGPTGSR	2806,3997	7,70
		GPAGPPGSTGFPGAAGR	1452,7109	0,00
		SAGISMPGPMGPMGPR	1541,7152	2,68
		GPPGPSGPPGLAGPPGEPGR	1749,8798	0,87
		GPSGPAGARGDK	1068,5311	0,53
		GQSGVMGFPGPK	1176,5597	0,21
		NGDRGETGPAGPAGPAGPAGAR	1931,9197	0,27
A0A2G9QE21	hemoglobin subunit β	VLNSIEEGLKHPENLK	1818,9839	9,13
		LLIVYPWTQR	1287,7339	6,44
		LGDVLIVTMAR	1186,6743	7,01
		VLNSIEEGLK	1100,6077	2,33
		GGSDVSAFLAK	1050,5345	0,56
		HSGELHVDPANFYR	1640,7695	0,22
Q8QGD4	monomeric α-macroglobulin	KGDSTVVIEDDSGFHR	1760,8329	0,00
		RLDEHATIEGETK	1497,7423	0,00
		QFIHVDDQDIQK	1484,7260	0,00
		DAQTKFDVHIEAR	1528,7633	0,00
A0A2G9R467	actin cytoplasmatic 2	SYELPDGQVITIGNER	1789,8846	27,23
		VAPEEHPVLLTEAPLNPK	1953,0570	0,00
		QEYDESGPSIVHR	1515,6953	0,00
		AVFPSIVGRPR	1197,6981	0,00
P21847	albumin	LLFLAHFTHDYAR	1602,8307	0,87
		EFPDIVFK	993,5171	0,00
		MPQVTAPTLVELAGR	1581,8549	0,00
		GLTLVQVSQKFGK	1403,8136	0,00
A0A385 KNQ9	vimentin-4	VEVDRDNMADDLQR	1674,7631	0,00
		FADLSEAANR	1092,5199	0,04
		KLESLQEEIIFLK	1588,9076	0,27
A5LHA5	annexin	DLVDDLKSELTGK	1431,7456	1,40
		VNDALVEQDAQDLFK	1703,8365	1,11
		GLGTDEDPIMK	1174,5540	0,13
C1C502	tropomyosin-1 α	IQLVEEELDR	1242,6455	0,27
		HIAEEADRKYEEVAR	1814,8911	0,02
		LVIIEGDLER	1155,6499	0,66
A0A2G9RE03	2-phospho-d-glycerate hydrolyase	AAVPSGASTGIYEALELRDNDKTR	2533,2771	0,00
		QIFDSRGNPTVEVDLFTAK	2136,0850	0,00
C1C500	histone H4	TVTAMDVVYALKR	1465,7963	0,00
		DNIQGITKPAIR	1324,7462	0,00
		VFLENVIR	988,5706	0,00
A0A2G9SLW0	IF rod domain	DLSLDGILR	1000,5553	5,16
		LADLEAALQK	1070,5972	0,00
A0A2G9SAK4	fibrinogen C-terminal	LIGTKPATSPQVIENPTQK	2021,1157	0,21
		ESVQIQEITGK	1230,6455	0,11
A0A2G9QD88	AhpC-TSA	QITINDLPVGR	1224,6826	0,25
		GLFIIDEKGILR	1372,8077	0,35
P55267	hemoglobin subunit α	VMNALTDAVK	1060,5587	0,06
		TYFPNFDFHANSAHLK	1907,8954	0,27
A0A2G9NCH1	collagen α-3 (VI) chain isoform X6	SAGSRIEEQVPQHLVLLTGGKSVDDVSGAAR	3175,6584	0,29
		VSELNTGDIEILR	1457,7726	1,36
		SIQSMTPIGGSTLNTGAALDYVQNNVFIGSAGSR	3425,6885	0,00
		DVVFLIDGSRDATPEFANVKELIGR	2760,4446	0,00
		SSDNIQAAANDLIR	1486,7375	0,00
		LLSSVTNLDQDSIKVIYENVPR	2502,3330	0,00
		IGTGVPQIAFIITGAK	1584,9238	1,29
		SSGVIPFAIGVR	1201,6819	0,00
		ELPNIESILFR	1329,7292	0,39
		VVEALDVDRDKIR	1526,8416	0,03
		VGLVQFSNDPTTEFFLK	1940,9883	0,00
A0A2G9P807	collagen α-1 (XII) chain isoform X3	LVEVFEIGPER	1286,6870	0,47
		NLNIYDIGTTTMR	1510,7450	1,53
A0A2G9RV90	collagen α-3 (VI) chain isoform X11	IVNRLEIGPDLIR	1506,8882	0,39
		ESVQIQEITGK	1230,6455	0,11

a IF: intermediate filament.

Following the identification of peptide sequences in the *F*4 fraction, molecular docking studies were performed to predict the intermolecular interactions between the peptides and COX-1 and COX-2. First, the analysis involved the 71 peptides listed in the previous table, aiming to elucidate their potential interaction with the COX-2 enzyme. Molecular docking analyses are crucial predictive tools that guide subsequent biological tests. By providing insights into molecular interactions, these studies enable better selection of therapeutic candidates and can accelerate the drug development process.^45,46^ Additionally, the findings from docking analyses often help mitigate unnecessary investments in time and resources, reducing the risk of failures in subsequent experimental phases. Consequently, docking analyses become essential for optimizing research and development strategies, contributing to more efficient and targeted advancements.^35,36,47^ Therefore, in our study, molecular docking was an important tool to evaluate the interactions between the selected peptides and the COX-2 enzyme (PDB ID: 5IKR). The COX-2 enzyme was selected as the molecular target due to its well-established role as a biomarker of inflammation and its relevance in the development of therapeutic agents with antiproliferative and anti-inflammatory properties.^48,49^ The predicted binding energies for the most stable complexes and the critical residues involved in the formation of the COX-2 inhibitor complex are summarized in Table 2. The energetic contribution of each residue within the catalytic site (≤4 Å) was evaluated, enabling the identification of key residues crucial for stabilizing the enzyme−ligand complex. Among the sequences evaluated, 11 exhibited significant affinity for COX-2, as detailed in Table 2. However, four peptides (SGHPGAMGPVGPR (P1), IGPAGSAGSR (P2), GPSGPAGARGDK (P3), and NGDRGETGPAGPAGPAGPAGAR (P4)) were selected for synthesis based on their higher binding energies and number of interactions within the COX-2 active site. In our study, docking analysis was highly important as a predictive tool, indicating which peptides could be considered potential candidates to present good results in biological studies. These results reduce the operational costs of development, increase success rates in identifying new therapeutic applicability for medicines, and optimize investment in laboratory analyses.^47^ In the past, the selection of new compounds for testing depended on the results obtained by chance, with opportune accidents enabling the identification of new applications for drugs originally intended for other therapeutic purposes.

**Table 2:** Binding Affinity Energy (kcal/mol) of the Interactions between the Ligands and COX-2 and the Peptide Sequences Along with the Number of Amino Acids in the COX-2 Active Site^a^

peptides	origin protein	COX-2 binding energy (kcal/mol)	number of amino acids in the active site
SGNRGEGGPSGPAGITGPSGPRGPAGPQGVR	collagen α-2 (I) chain	−6,9	4
SGHPGAMGPVGPR		−9,5	3
GPSGPSGPPGKEGR		0	0
GAPGERGEAGPAGPTGFAGPPGAAGHTGAKGDR		0	0
IGPAGSAGSR		−8,8	8
AGGIGPAGSR		0	0
GIPGPAGPAGPSGAR		0	0
GPAGAAGAPGPAGGPGDRGESGPAGPSGVAGPR		0	0
SLNQQIEVILTPEGSRK		0	0
GLTGPIGPPGPGGAPGDKGEAGPAGPAGPTGSR	collagen α-1 (I) chain	0	0
GPAGPPGSTGFPGAAGR		−11	1
SAGISMPGPMGPMGPR		-	-
GPPGPSGPPGLAGPPGEPGR		−9,1	1
GPSGPAGARGDK		−11	4
GQSGVMGFPGPK		0	0
NGDRGETGPAGPAGPAGPAGAR		−10,7	9
VLNSIEEGLKHPENLK	hemoglobin subunit β	−6,3	1
LLIVYPWTQR		−7,2	7
LGDVLIVTMAR		−7,7	3
VLNSIEEGLK		0	0
GGSDVSAFLAK		−7,4	2
HSGELHVDPANFYR		−6,4	3
KGDSTVVIEDDSGFHR	monomeric α-macroglobulin	0	0
RLDEHATIEGETK		−6,2	8
QFIHVDDQDIQK		0	0
DAQTKFDVHIEAR		−6,5	6
SYELPDGQVITIGNER	actin cytoplasmatic 2	−6,6	1
VAPEEHPVLLTEAPLNPK		0	0
QEYDESGPSIVHR		0	0
AVFPSIVGRPR		−7,5	3
LLFLAHFTHDYAR	albumin	0	0
EFPDIVFK		−6,7	2
MPQVTAPTLVELAGR		−5,5	2
GLTLVQVSQKFGK		−6,3	2
VEVDRDNMADDLQR	vimentin-4	0	0
FADLSEAANR		0	0
KLESLQEEIIFLK		−5,4	2
DLVDDLKSELTGK	annexin	0	0
VNDALVEQDAQDLFK		0	0
GLGTDEDPIMK		0	0
IQLVEEELDR	tropomyosin-1 α	−8,6	1
HIAEEADRKYEEVAR		−5,5	2
LVIIEGDLER		−6,1	3
AAVPSGASTGIYEALELRDNDKTR	2-phospho-d-glycerate hydrolyase	0	0
QIFDSRGNPTVEVDLFTAK		−6,6	7
TVTAMDVVYALKR	histone H4	−6,1	3
DNIQGITKPAIR		0	0
VFLENVIR		0	0
DLSLDGILR	IF rod domain	−7,9	1
LADLEAALQK		−7,1	4
LIGTKPATSPQVIENPTQK	fibrinogen C-terminal	0	0
ESVQIQEITGK		−7,3	2
QITINDLPVGR	AhpC-TSA	−7,8	4
GLFIIDEKGILR		−6,9	2
VMNALTDAVK	hemoglobin subunit α	−7,7	4
TYFPNFDFHANSAHLK		−7,7	1
SAGSRIEEQVPQHLVLLTGGKSVDDVSGAAR	collagen α-3 (VI) chain isoform X6	0	0
VSELNTGDIEILR		−6,5	1
SIQSMTPIGGSTLNTGAALDYVQNNVFIGSAGSR		-	-
DVVFLIDGSRDATPEFANVKELIGR		−6,3	1
SSDNIQAAANDLIR		−6,2	5
LLSSVTNLDQDSIKVIYENVPR		−6,6	8
IGTGVPQIAFIITGAK		-	-
SSGVIPFAIGVR		−6,8	1
ELPNIESILFR		0	0
VVEALDVDRDKIR		−7,3	3
VGLVQFSNDPTTEFFLK		−6,9	8
LVEVFEIGPER	collagen α-1 (XII) chain isoform X3	−7,8	1
NLNIYDIGTTTMR		0	0
IVNRLEIGPDLIR	collagen α-3 (VI) chain isoform X11	0	0
ESVQIQEITGK		−7,3	2

a The peptides highlighted in gray (SGHPGAMGPVGPR; IGPAGSAGSR; GPSGPAGARGDK; NGDRGETGPAGPAGPAGPAGAR; LLIVYPWTQR; LGDVLIVTMAR; AVFPSIVGRPR; LADLEAALQK; QITINDLPVGR; VMNALTDAVK; VVEALDVDRDKIR) exhibited the highest affinity for cyclooxygenase-2 (COX-2), as determined by molecular docking analysis.

The accommodation of peptides within the active site of cyclooxygenase-2 (COX-2) is illustrated in Figure 2, highlighting the amino acid residues involved in the interactions. The analysis of these interactions is crucial for elucidating the inhibitory potential of the peptides and their affinity for the enzyme. Figure 2A shows that the COX-2 catalytic site contains key residues, including tyrosine 355 (TYR355), leucine 359 (LEU359), valine 523 (VAL523), phenylalanine 381 (PHE381), and tryptophan 381 (TRP381), which contribute to substrate stabilization and target inhibitory interactions. As shown in Figure 2B–E, we observed that the amino acid residue peptides also contained the same amino acids, justifying the strong affinity of the peptides for COX-2. It is essential to highlight that leucine 359 (LEU359) facilitates substrate positioning within the active site, while valine 523 (VAL523), situated near the entrance of the catalytic site, may influence peptide accommodation. Additionally, the aromatic residues phenylalanine 381 (PHE381) and tryptophan 381 (TRP381) can engage in π−π stacking interactions with peptides containing aromatic rings, affecting the binding stability and affinity. However, despite the similarity, the four analyzed peptides (P1 to P4) exhibited different interaction patterns with the COX-2 active site, suggesting distinct mechanisms of enzyme−ligand complex stabilization. Peptide P1 interacted with TYR355, LEU359, PHE381, and VAL523. Its structural rigidity, conferred by proline (P), may enhance interaction stability, particularly through hydrophobic forces and π−π stacking with PHE381. Peptide P2 strongly interacted with TYR355 and VAL523, with potential arginine (R) contributions in establishing hydrogen bonds and ionic interactions. In the case of peptide P3, predominant interactions occurred with VAL523 and LEU359, influenced by electrostatic interactions due to aspartic acid (D) and lysine (K). Additionally, the repetition of proline may impact the peptide’s conformation within the active site. Peptide P4, on the other hand, exhibited strong interactions with TYR355 and LEU359, characterized by a high number of glycine (G) and proline (P) residues, which provide greater structural flexibility. Additionally, possible ionic interactions were mediated by glutamic acid (E) and arginine (R). The affinity for TYR355 observed in some peptides suggests a potential inhibitory effect on COX-2, as it may hinder the accommodation of the natural substrate. Furthermore, the stabilization of the complex depends on the balance between hydrophobic interactions (PHE381 and LEU359) and electrostatic interactions (ARG, GLU, and ASP). The binding pattern of these peptides may be a crucial determinant of COX-2 selectivity over COX-1, a fundamental factor in developing selective inhibitors with therapeutic potential.^50^ Although COX-1 and COX-2 share active sites composed of similar amino acid groups, a critical structural difference exists: the residue at position 523, which is an isoleucine in COX-1 and a valine in COX-2.^51^ This variation may affect interactions with inhibitors, influencing their specificity and efficacy.

**Figure 2:**
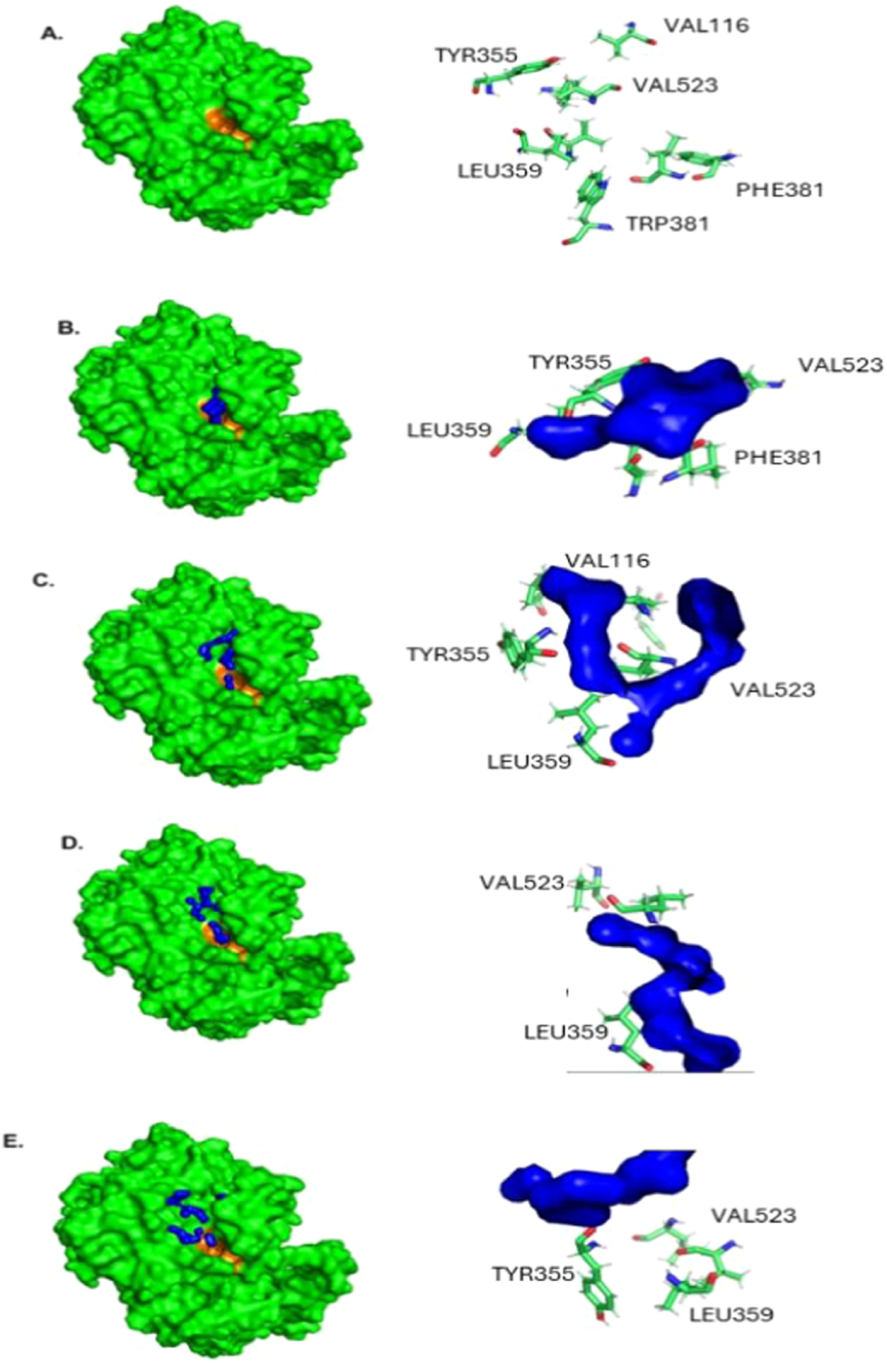
Active site and peptide interactions. Cyclooxygenase-2 (green) enzyme with its catalytic site (orange color) (A). The blue loops represent the location of the peptides and the amino acid residues that compose them. Interaction of the COX-2 active site with the peptides SGHPGAMGPVGPR (P1) (B), IGPAGSAGSR (P2) (C), GPSGPAGARGDK (P3) (D), and NGDRGETGPAGPAGPAGPAGAR (P4) (E).

Thinking about the different interactions between COX-1 and COX-2, the next step was to evaluate whether the four peptides exhibited selective COX-2 inhibition. Therefore, molecular docking was also performed with the cyclooxygenase-1 enzyme (COX-1) (PDB ID: 6Y3C). The interactions revealed variations in the binding patterns of the peptides between isoforms, suggesting possible differences in the accessibility or arrangement of residues within the active sites. As listed in Table 3, it was possible to observe that the peptides showed a high affinity for COX-1, as evidenced by the inhibition energy values and the number of amino acids involved in the interaction. These results indicate the potential of the peptides as enzyme modulators, highlighting their possible applications in various biological and therapeutic contexts. Peptides can act as competitive or allosteric inhibitors, blocking the conversion of arachidonic acids to prostaglandins (PGs). COX-1 catalyzes the oxygenation of arachidonic acid to form PGG2, which is subsequently converted into PGH2. Prostaglandins, such as PGE2, are derived from this pathway and are directly involved in regulating inflammatory processes.^52^ Peptides with charged residues, such as arginine and aspartic acid, can interfere with the binding of arachidonic acid to the hydrophobic active site of COX-1, preventing its conversion into proinflammatory intermediates. Additionally, peptides that stabilize alternative enzyme conformations can induce allosteric changes that reduce catalytic activity, even without directly blocking the active site.^53^ In terms of signaling pathways, the reduction in prostaglandin synthesis via COX-1 inhibition affects key molecular cascades. The decrease in PGE2 reduces the activation of G-protein-coupled prostaglandin receptors, attenuating the activation of pathways like PKA and PI3K/AKT, which promote the expression of proinflammatory genes via NF-κB. Thus, COX-1 inhibition can modulate the release of cytokines such as IL-6 and TNF-α, reducing the amplification of the inflammatory response.^54^ Therefore, peptides derived from bullfrog skin emerge as promising candidates for regulating the COX-1 activity through multiple mechanisms, either by directly interfering with the active site or by promoting allosteric conformational changes. This makes them potent inflammation modulators, with potential for the development of new selective anti-inflammatory therapies that are less harmful to the body’s homeostatic balance.

After analyzing the molecular docking results for COX-2 and COX-1, we observed that all peptides tested exhibit good affinity for both enzymes. This dual inhibition may offer therapeutic benefits, including faster alleviation of pain and inflammation compared with compounds that selectively inhibit only one of these enzymes. In addition, simultaneous COX-1 and COX-2 inhibition has been associated with regulating cell and vascular proliferation, contributing to the control of chronic inflammation.^55–57^ Among the four peptides, it is possible to highlight peptide 3 (GPSGPAGARGDK) with a binding energy of −11 kcal/mol for COX-2 and −9.7 kcal/mol for COX-1, suggesting that it is the most promising candidate for selective inhibition of these isoforms. Its high binding potential to COX-2 and COX-1 indicates that this peptide could be used as a solid referential basis for developing new compounds with enhanced selectivity.^58–60^ However, although docking methods are powerful in predicting molecular interactions, they assume ideal conditions that do not always reflect actual cellular or physiological conditions. Therefore, it is important to gain biological insights to prove the results presented in the molecular docking results.

**Table 3:** Binding Affinity Energy of the Interactions between the Ligands and COX-1 and the Number of Amino Acids in the Active Site Located within 4 Å, *i.e*., the Amino Acids Closest to the Catalytic Site and the Ligand

peptides	COX-1 binding energy (kcal/mol)	number of amino acids in the active site
P1-SGHPGAMGPVGPR	−9,5	6
P2-IGPAGSAGSR	−9,5	4
P3-GPSGPAGARGDK	−9,7	4
P4-NGDRGETGPAGPAGPAGPAGAR	−9,5	6

In our study, the next step was to make an *in vitro* proof of concept to allow the identification of less toxic and more efficient and relevant peptides to be used in future drug development. For this, cell viability assays revealed that P1, P2, P3, and P4 exhibited no cytotoxic effects (Figure 3), with the viability percentage remaining close to 100%. The absence of cytotoxicity is the first and most important characteristic to measure potential therapeutic applications for new compounds.^61,62^ Excessive cytotoxicity could limit the therapeutic applicability of a compound, whereas low cytotoxicity increases its potential for successful clinical translation. Our next step was to follow with *in vitro* proof of concept through the total COX quantification. Surprisingly, only peptide P1 exhibited an inhibitory capacity on the total COX activity, at a concentration of 1 mM compared to the other groups, which may be related to specific interactions within its active site (Figure 4). Based on these results, it is interesting to highlight that the molecular docking results were not exactly the same as in the *in vitro* proof of concept, but the docking analyses were important to give us an orientation to choose the four peptides and give us a good prediction for a possible biological effect. Actually, the docking model used may not have considered factors such as the flexibility of the protein and peptide, interactions with other molecules, or the effect of the solvent on the behavior of the molecule. This type of restriction is common, as docking often works with rigid structures and with few considerations about molecular dynamics. *In vitro* experiments, in turn, involve a series of additional factors that may not be considered in computational simulations. The presence of cofactors, cellular dynamics, and the three-dimensional conformation of proteins in an aqueous or cellular environment and the possibility of unforeseen effects in the experimental environment can influence interactions unpredictably. Furthermore, dosing methods, incubation time, peptide concentrations, and other experimental parameters can affect results, leading to differences in observed effects compared to computational variations.

**Figure 3:**
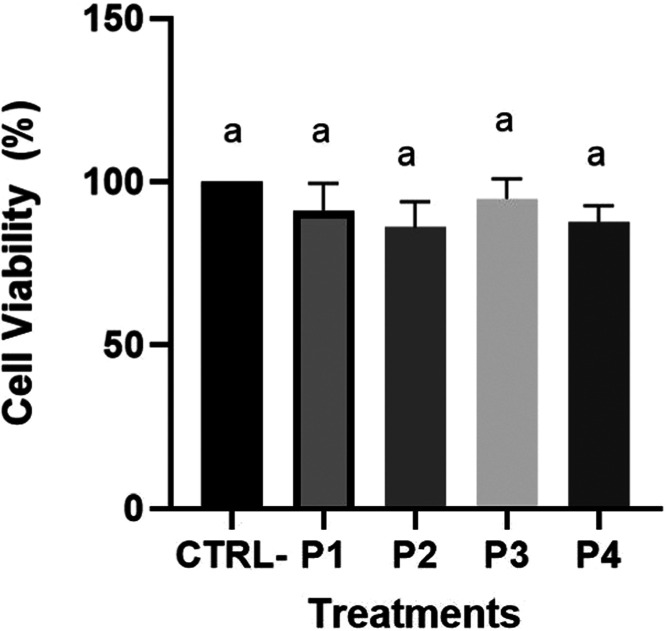
Effect of the peptides SGHPGAMGPVGPR (P1), IGPAGSAGSR (P2), GPSGPAGARGDK (P3), and NGDRGETGPAGPAGPAGPAGAR (P4) on the viability of the RAW 264.7 macrophages. Cells were cultured in RPMI and treated with each peptide at a concentration of 1 mM. The negative control group consisted of cells cultured in DMEM without peptide treatment. Data (*n* = 4) are expressed as mean ± standard error of the mean. Means followed by the same letter are not significantly different (*p* > 0.05) by Tukey’s post hoc test.

**Figure 4:**
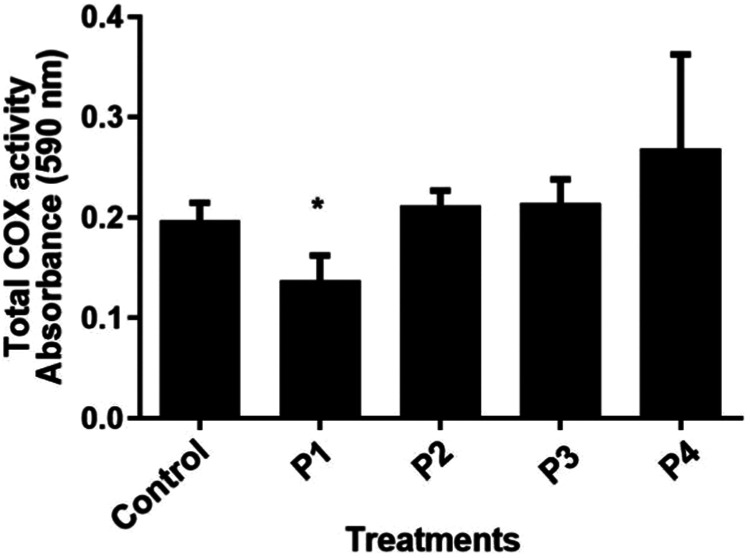
Effect of SGHPGAMGPVGPR (P1), IGPAGSAGSR (P2), GPSGPAGARGDK (P3), and NGDRGETGPAGPAGPAGPAGAR (P4) peptides on the total COX activity. The RAW 264.7 cells were subcultured at 1 × 10^8^ cells/mL density and stimulated with LPS at 1 μg/mL for 4 h. Cells were collected by centrifugation at a speed of 2.000 rpm for 10 min at 4 °C and were resuspended in DMEM. After that, cells were treated with the peptides, and ELISA was performed. Significant by the *t* test (*P* < 0.05). CTRL: control (cells stimulated with LPS).

Since only P1 demonstrated the ability to reduce the total COX activity, different concentrations (0.025, 0.05, 0.5, and 1 mM) of P1 were tested to assess their effects on the IL-6 concentration. The results indicated that 1 mM P1 was the only treatment that significantly downregulated IL-6 levels in a dose-dependent manner (Figure 5), reinforcing our biological results obtained for the total COX activity. Therefore, we can observe that peptide 1 has a great capacity to control the inflammation process. Given the direct impact of COX-2 inhibition on inflammatory mediators such as IL-6,^63,64^ it was essential to assess whether this biochemical effect would translate into a measurable cellular response. Interleukin 6 (IL-6) is a cytokine essential for maintaining homeostasis.^65^ When homeostasis is disrupted by infections or tissue injuries, IL-6 is rapidly produced and plays an important role in defending the organism by activating the acute phase and immune responses. However, excessive and prolonged production of IL-6 can have pathological effects, such as in the acute systemic inflammatory response syndrome and chronic immune-mediated diseases.^66^ Therefore, identifying compounds capable of inhibiting this pathway is crucial for developing effective treatments.^67–69^ These findings underscore the importance of optimizing the concentration of P1 to maximize its therapeutic potential. The combined data from COX assays and IL-6 analysis identify peptide 1 as a promising candidate for anti-inflammatory interventions.

**Figure 5:**
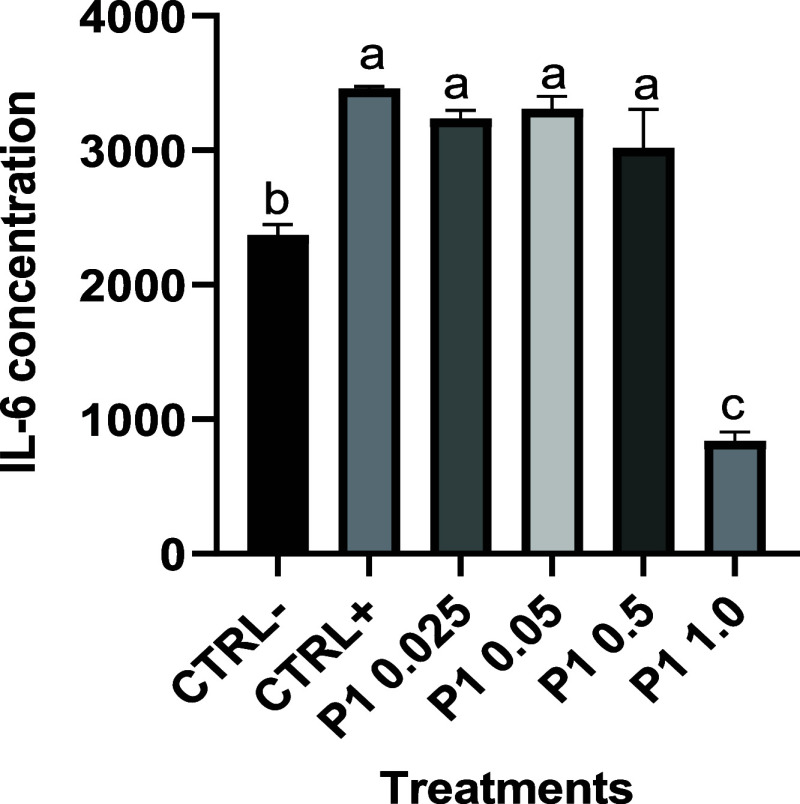
IL-6 concentration (pg/mL) in macrophages treated at 0.025, 0.05, 0.5, and 1.0 mM peptide 1 and exposure to 1 μg/mL LPS for 4 h. Ctrl-: Negative control (cells + DMEM); Ctrl+: Positive control 1 (cells + DMEM+ and LPS); P1 (cells + DMEM + peptide at different concentrations + LPS). Data (*n* = 3) are expressed as mean and standard error. Means followed by the same letter are not significantly different (*p* > 0.05) by Tukey’s post hoc test.

## 6 Conclusions

This study demonstrated the promising therapeutic potential of the four peptides, especially peptide 1 (SGHPGAMGPVGPR P1), in modulating the inflammatory response. Molecular docking analysis revealed favorable interactions between the 4 peptides and the COX-2 and COX-1 active sites, suggesting the high affinity and selectivity of peptide 3 (GPSGPAGARGDK - P3), which may explain its effectiveness in inhibiting COX. However, the biological proof of concept showed that peptide 1 (P1) presented the best results in decreasing the cells’ total COX and IL-6 cytokines. Although some divergence was presented between molecular docking and *in vitro* findings (biological proof), our study showed that integrative analyses are very important for developing new therapeutic targets, increasing success rates in identifying new therapeutic applicability. Controlled studies are required to determine whether and to what extent additional mechanisms contribute in controlling the inflammatory process after these peptide exposures, especially peptide 1.

## Supplementary Material



## Notes

Chromatograms, mass spectra (MS^1^ and MS^2^), and ion tables of four peptides identified by molecular docking (SGHPGAMGPVGPR, IGPAGSAGSR, GPSGPAGARGDK, NGDRGETGPAGPAGPAGPAGAR) and HPLC analyses and peptide sequences obtained from AminoTech (PDF)

## Abbreviations

COX-1: cyclooxygenase-1
COX-2: cyclooxygenase-2
CTRL: control
*F*1: fraction 1
*F*2: fraction 2
*F*3: fraction 3
*F*4: fraction 4
IL-6: interleukin 6
LEU: leucine
NFκB: nuclear factor kappa B
P1: peptide 1 SGHPGAMGPVGPR
P2: peptide 2 IGPAGSAGSR
P3: peptide 3 GPSGPAGARGDK
P4: peptide 4 NGDRGETGPAGPAGPAGPAGAR
PGE: prostaglandin
PHE: phenylalanine
TNFα: tumor necrosis factor α
TRP: tryptophan
TYR: tyrosine
VAL: valine
