# Supplementary material for: Modulation of the Inflammatory Process through the Control of Cyclooxygenase Using Peptides Obtained from Lithobates Catesbeianus Identified by Molecular Docking
Source: ACS Omega. 2025 May 14;10(20):20830–43. doi: 10.1021/acsomega.5c02159 (PMC12120644; doi:10.1021/acsomega.5c02159)
Supplement: Supplementary file 1 [file ao5c02159_si_002.pdf]

SUPPLEMENTARY MATERIAL

Modulation of the Inflammatory Process Through the Control of Cyclooxygenase Using Peptides Obtained From *Lithobates catesbeianus* Identified by Molecular Docking

Patricia da Silva Mattosinhos[1], Silvania Mol Pelinsari[2], Raul Santos Alves[3], Manoela Maciel dos Santos Dias[4], Rômulo Dias Novaes[5], Edvaldo Barros[6], Marcos Rogério Tótola[7], Tiago de Oliveira Mendes[8], Reggiani Vilela Gonçalves[1],[9]*

[1] Department of General Biology, Federal University of Viçosa, Viçosa, MG, Brazil

[2] Department of General Biology, Federal University of Viçosa, Viçosa, MG, Brazil

[3] Department of General Biology, Federal University of Viçosa, Viçosa, MG, Brazil

[4] Department of Animal Biology, Federal University of Viçosa, Viçosa, MG, Brazil

[5] Department of Structural Biology, Federal University of Alfenas, Alfenas, MG, Brazil

[6] Biomolecule Analysis Center (NUBIOMOL), Federal University of Viçosa, Viçosa, MG, Brazil

[7] Department of Microbiology, Federal University of Viçosa, Viçosa, MG, Brazil

[8] Department of Biochemistry and Molecular Biology, Federal University of Viçosa, Viçosa, MG, Brazil

[9] Department of Animal Biology, Federal University of Viçosa, Viçosa, MG, Brazil; Plants for Human Health Institute, North Carolina Research Campus, Kannapolis, NC, USA

* Corresponding author: Reggiani Vilela Gonçalves

E-mail: reggysvilela@yahoo.com.br

Tel.: +55-31-3612-5251

Fax: +55-31-3612-5252

Appendix 1:

Chromatogram of peptide 1

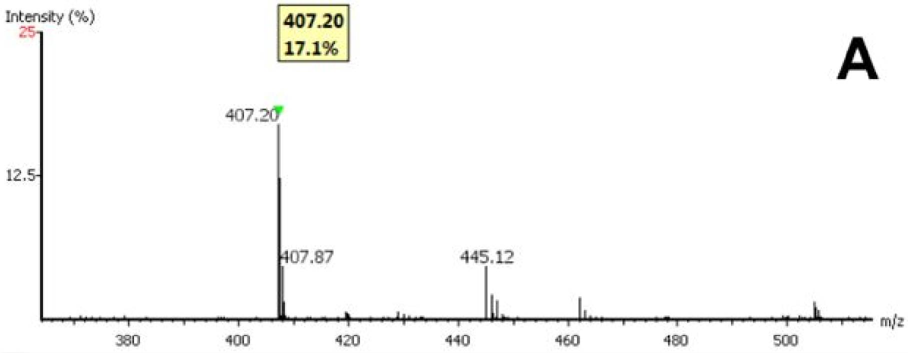

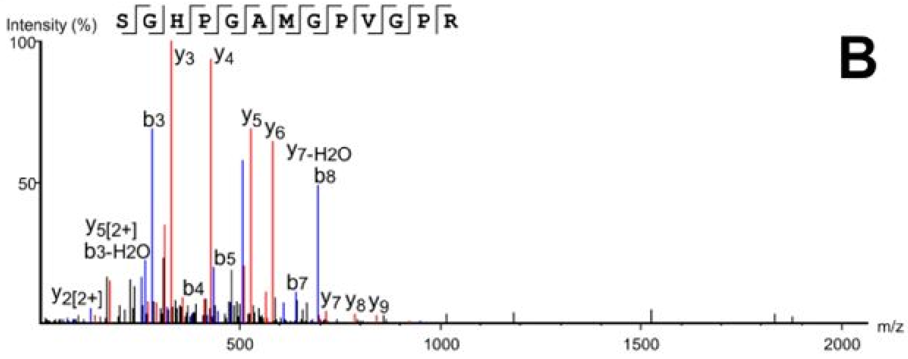

C

#	b	b-H2O	b-NH3	b (2+)	Seq	y	y-H2O	y-NH3	y (2+)	#
1	87.86	70.55	70.55	44.52	S					13
2	145.06	127.08	128.03	73.03	G	1132.57	1114.56	1115.54	567.30	12
3	282.12	264.11	265.09	141.56	H	1075.55	1057.54	1058.52	538.27	11
4	379.19	361.16	362.15	190.09	P	938.49	920.48	921.49	469.74	10
5	436.19	418.21	419.17	218.60	G	841.43	823.42	824.41	421.22	9
6	507.24	489.22	490.20	254.15	A	784.41	766.40	767.39	392.71	8
7	638.28	620.26	621.25	320.14	M	713.38	695.29	696.30	357.16	7
8	695.29	677.28	678.27	348.15	G	582.33	564.26	565.31	292.15	6
9	792.35	774.34	775.32	396.67	P	525.32	507.24	508.25	263.67	5
10	891.41	873.40	874.39	446.21	V	428.26	410.25	411.24	214.63	4
11	948.43	930.43	931.41	474.21	G	329.19	311.17	312.17	165.10	3
12	1045.49	1027.48	1028.46	523.24	P	272.17	254.15	255.14	136.59	2
13					R	175.12	157.11	158.09	87.86	1

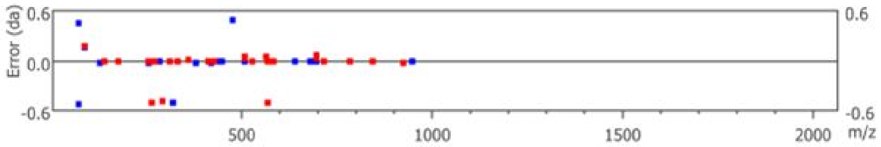

A - Mass spectrum MS1 of the peptide SGHPGAMGPVGPR;

B - Mass spectrum MS2 of the peptide SGHPGAMGPVGPR;

C - Ions table of te peptide SGHPGAMGPVGPR.

Chromatogram of peptide 2

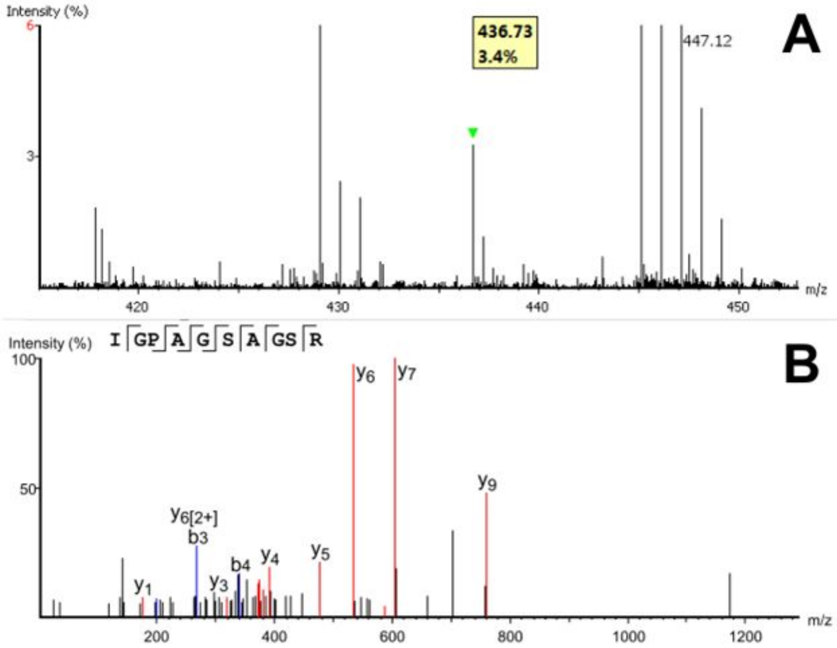

C

#	b	b-H2O	b-NH3	b (2+)	Seq	y	y-H2O	y-NH3	y (2+)	#
1	114.09	96.08	97.06	57.55	I					10
2	171.11	153.10	154.09	86.06	G	759.38	741.36	742.35	380.19	9
3	268.17	250.16	251.14	134.58	P	702.35	684.34	685.33	351.68	8
4	339.11	321.19	322.18	170.10	A	605.30	587.29	588.27	303.15	7
5	396.22	378.21	379.20	199.19	G	534.26	516.25	517.24	268.17	6
6	483.26	465.25	466.23	242.13	S	477.24	459.23	460.21	239.12	5
7	554.29	536.28	537.27	277.65	A	390.21	371.70	373.20	195.60	4
8	611.32	593.30	594.29	306.16	G	319.17	301.16	302.15	160.09	3
9	698.35	680.34	681.32	349.67	S	262.15	244.14	245.12	131.58	2
10					R	175.12	157.11	158.09	88.06	1

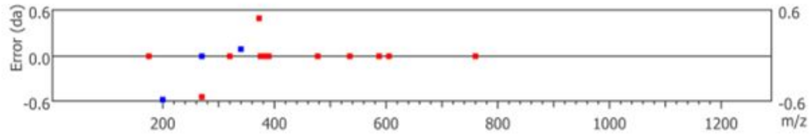

A - Mass spectrum MS1 of the peptide IGPAGSAGSR;

B - Mass spectrum MS2 of the peptide IGPAGSAGSR;

C - Ions table of te peptide IGPAGSAGSR.

Chromatogram of peptide 3

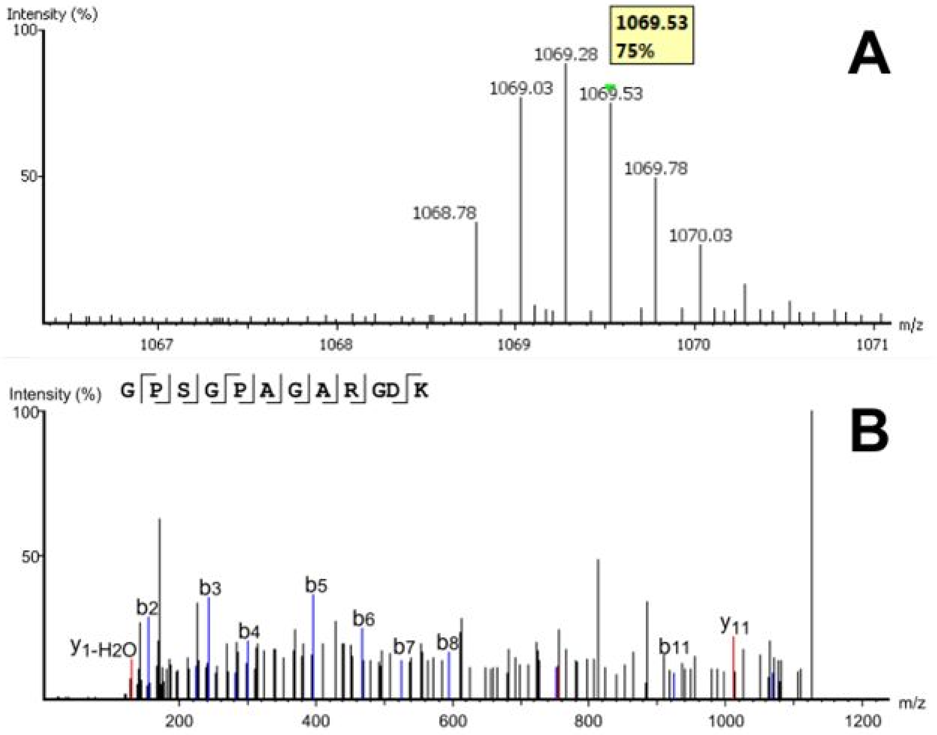

C

#	b	b-H2O	b-NH3	b (2+)	Seq	y	y-H2O	y-NH3	y (2+)	#
1	58.03	40.02	41.00	29.51	G					12
2	155.08	137.07	138.06	78.04	P	1012.54	994.51	995.49	506.76	11
3	242.12	224.11	225.09	121.56	S	915.46	897.45	898.44	458.23	10
4	299.15	281.13	282.11	150.07	G	828.43	810.42	811.41	414.72	9
5	396.19	378.18	379.16	198.59	P	771.41	753.38	754.38	386.21	8
6	467.22	449.21	450.20	234.11	A	674.36	656.35	657.33	337.68	7
7	524.25	506.24	507.22	262.62	G	603.32	585.31	586.29	302.16	6
8	595.30	577.27	578.26	298.14	A	546.30	528.29	529.27	273.65	5
9	751.37	733.37	734.36	376.19	R	475.26	457.25	458.24	238.13	4
10	808.41	790.40	791.38	404.70	G	319.16	301.15	302.13	160.08	3
11	923.44	905.42	906.41	462.22	D	262.14	244.13	245.11	131.57	2
12					K	147.11	129.10	130.09	74.06	1

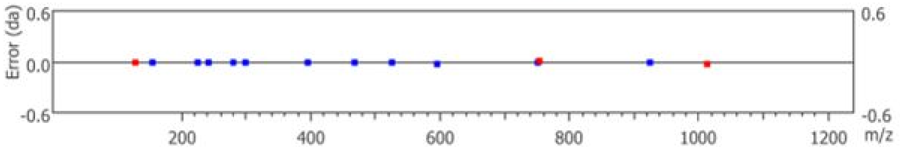

A - Mass spectrum MS1 of the peptide GPSGPAGARGDK;

B - Mass spectrum MS2 of the peptide GPSGPAGARGDK;

C - Ions table of te peptide GPSGPAGARGDK.

Chromatogram of peptide 4

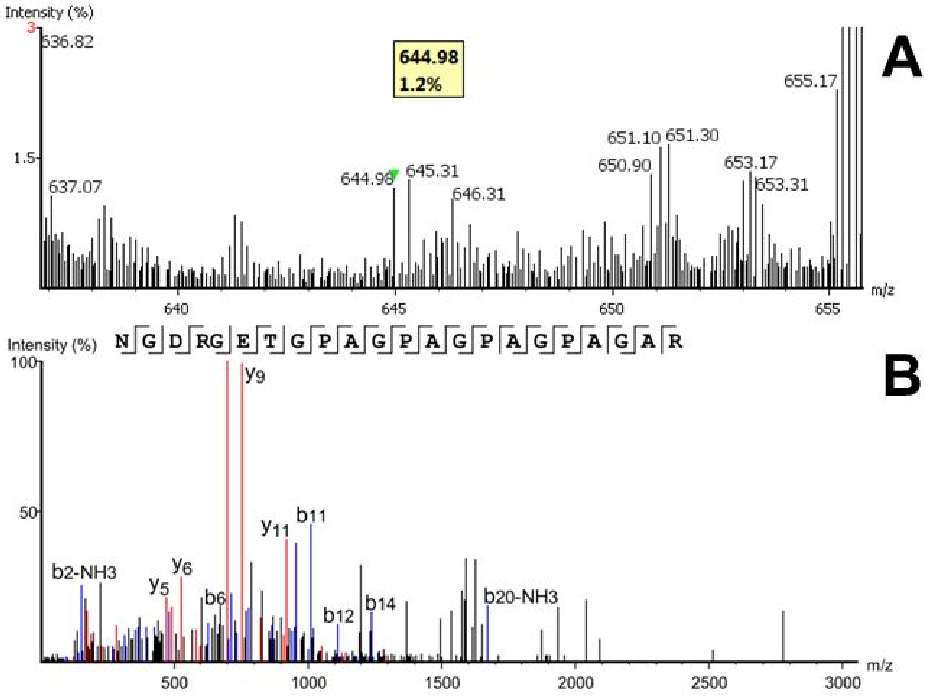

C

#	b	b-H2O	b-NH3	b (2+)	Seq	y	y-H2O	y-NH3	y (2+)	#
1	115.05	97.37	98.02	58.03	N					22
2	172.07	154.06	155.08	86.57	G	1818.88	1800.87	1801.86	910.43	21
3	287.10	269.09	270.12	144.10	D	1761.86	1743.85	1744.84	881.43	20
4	443.20	425.19	426.17	222.10	R	1646.84	1628.83	1629.81	824.41	19
5	500.22	482.21	483.22	250.61	G	1490.73	1472.72	1473.71	745.87	18
6	629.27	611.25	612.24	315.18	E	1433.71	1415.70	1416.69	717.36	17
7	730.31	712.33	713.29	366.21	T	1304.67	1286.56	1287.64	652.84	16
8	787.33	769.32	770.31	394.20	G	1203.62	1185.61	1186.60	602.31	15
9	884.39	866.43	867.36	442.69	P	1146.59	1128.50	1129.57	573.80	14
10	955.44	937.41	938.43	478.21	A	1049.55	1031.54	1032.52	525.27	13
11	1012.45	994.43	995.42	506.72	G	978.51	960.50	961.48	489.75	12
12	1109.52	1091.49	1092.47	555.25	P	921.48	903.48	904.46	461.24	11
13	1180.54	1162.52	1163.51	590.77	A	824.41	806.43	807.41	412.72	10
14	1237.55	1219.55	1220.53	619.28	G	753.40	735.39	736.37	377.20	9
15	1334.61	1316.60	1317.58	667.80	P	696.38	678.37	679.35	348.69	8
16	1405.65	1387.64	1388.62	703.30	A	599.23	581.32	582.25	300.16	7
17	1462.67	1444.66	1445.64	731.68	G	528.29	510.28	511.26	264.64	6
18	1559.72	1541.71	1542.69	780.37	P	471.26	453.26	454.24	236.13	5
19	1630.76	1612.75	1613.73	815.88	A	374.21	356.20	357.19	187.07	4
20	1687.78	1669.77	1670.76	844.39	G	303.18	285.17	286.16	152.09	3
21	1758.82	1740.81	1741.79	879.91	A	246.16	228.12	229.13	123.88	2
22					R	175.12	157.11	158.09	88.06	1

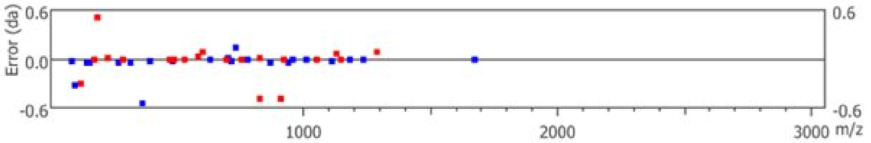

A - Mass spectrum MS1 of the peptide NGDRGETGPAGPAGPAGPAGAR;
B - Mass spectrum MS2 of the peptide NGDRGETGPAGPAGPAGPAGAR;
C - Ions table of te peptide NGDRGETGPAGPAGPAGPAGAR.

Appendix 2:
HPLC Analysis of four peptides – Adquired by AminoTech

Peptide 1: SGHPGAMGPVGPR

Análise por Espectrometria de Massas

NH_2-Ser-Gly-His-Pro-Gly-Ala-Met-Gly-Pro-Val-Gly-Pro-Arg-COOH
(L:23112)

Model: LCMS-2020	Entrance Lens: +20,0 V	M	1219,3
RF Gain: 5000	Interface: ESI	$[M+H]^{+}$	1220,3
RF Offset: 5100	Nebulizing Gas Flow: 1,50 L/min	$[M+2H]^{+2}$	610,7
Mainrod Bias: +5,0 V	Drying Gas Flow: 15,00 L/min	$[M+3H]^{+3}$	407,4
Conversion Dynode: +10,0 kV	Interface Bias: -3,50 kV	$[M+4H]^{+4}$	305,8
Detector: -1,10 kV	Interface Bias: -3,50 kV	$[M+5H]^{+5}$	244,9
PG: 0,0e+000 Pa	Interface Current: 0,0 uA	$[M+6H]^{+6}$	204,2
IG: 0,0e+000 Pa	Heat Block Temp.: 150 C		
DL Temp.: 150 C			

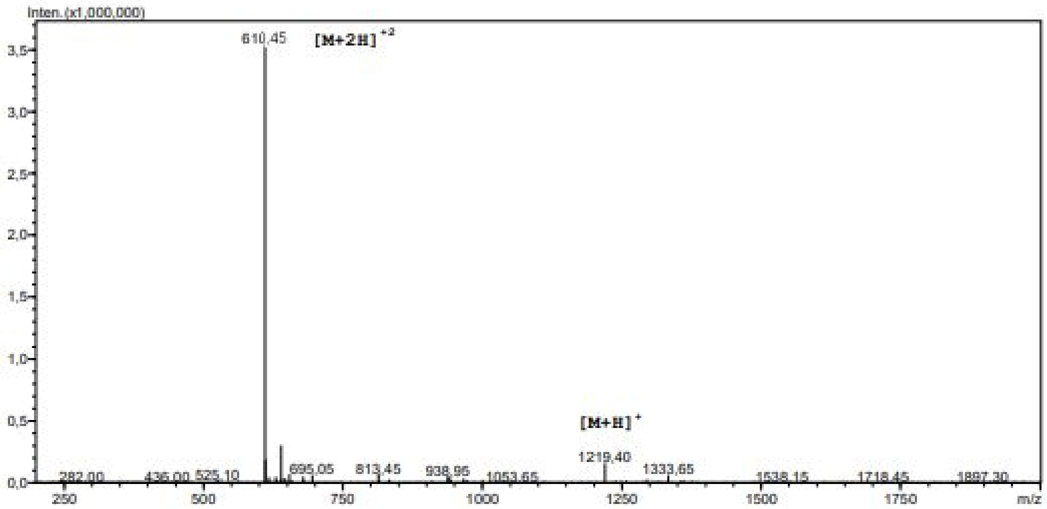

Peptide 2: IGPAGSAGSR

AMINOTECH PESQUISA E DESENVOLVIMENTO - ANÁLISE POR HPLC

C:\LabSolutions\Data\Project1\23113R-LCF02.lcd

Acquired by	: System Administrator
Sample Name	: 23113R-LCF
Sample ID	: 23113R-LCF
Tray#	: 1
Vial#	: 1
Injection Volume	: 20
Data File	: C:\LabSolutions\Data\Project1\23113R-LCF02.lcd
Method File	: C:\LabSolutions\Data\Project1\AmT-AnalCOLETA-ISO.lcm
Report Format File	: C:\LabSolutions\System\DEFAULT.lsr
Month-Day Acquired	: 20/11/2023
Month-Day Processed	: 20/11/2023

NH2-Ile-Gly-Pro-Ala-Gly-Ser-Ala-Gly-Ser-Arg-COOH

C:\LabSolutions\Data\Project1\23113R-LCF02.lcd

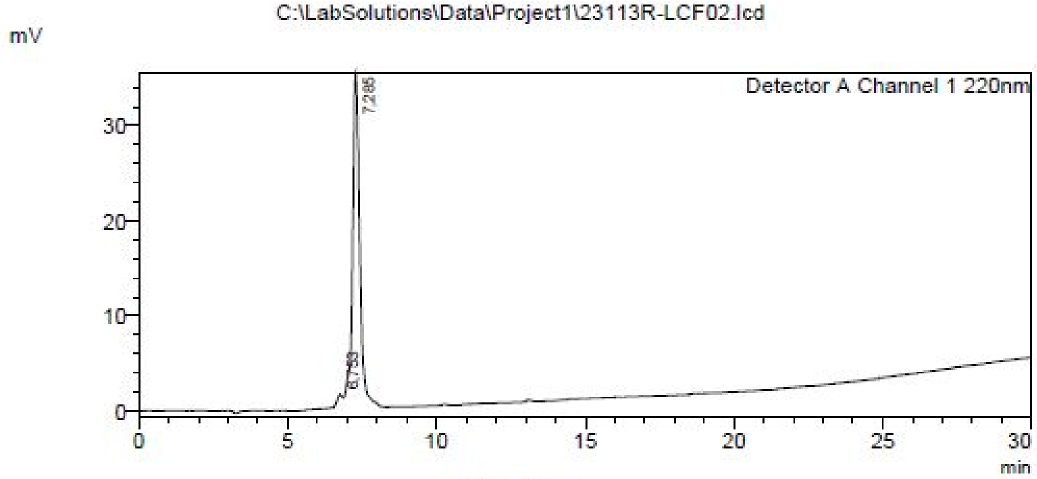

Peak Table

Detector A Channel 1 220nm

Peak#	Ret. Time	Area	Area%	Height	Height%	Conc.	Mark
1	6,753	14648	2,495	1184	3,287	2,495	
2	7,285	572458	97,505	34832	96,713	97,505	V
Total		587106	100,000	36016	100,000		

Condições de Análise
Coluna RP-C18, 4,6x250mm - Fluxo: 1mL/min
Solventes: A=TFA 0.1%/H2O. B=CH3CN/H2O 9:1 com TFA 0.1%

Peptide 3: GPSGPAGARGDK

AMINOTECH PESQUISA E DESENVOLVIMENTO - ANÁLISE POR HPLC

C:\LabSolutions\Data\Project1\23114R-LCF01.lcd

Acquired by	: System Administrator
Sample Name	: 23114R-LCF
Sample ID	: 23114R-LCF
Tray#	: 1
Vial#	: 1
Injection Volume	: 20
Data File	: C:\LabSolutions\Data\Project1\23114R-LCF01.lcd
Method File	: C:\LabSolutions\Data\Project1\AmT-AnalCOLETA-ISO.lcm
Report Format File	: C:\LabSolutions\System\DEFAULT.lsr
Month-Day Acquired	: 20/11/2023
Month-Day Processed	: 20/11/2023

NH2-Gly-Pro-Ser-Gly-Pro-Ala-Gly-Ala-Arg-Gly-Asp-Lys-COOH

C:\LabSolutions\Data\Project1\23114R-LCF01.lcd

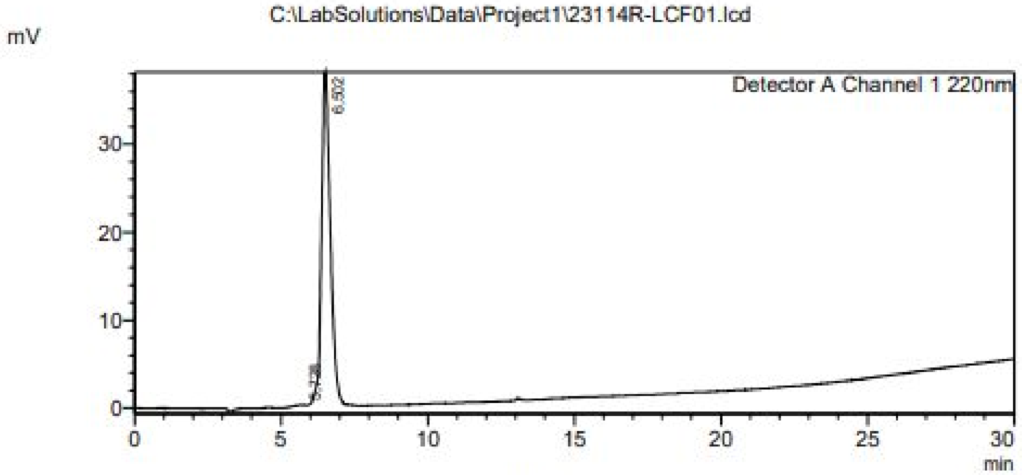

Peak Table

Detector A Channel 1 220nm

Peak#	Ret. Time	Area	Area%	Height	Height%	Conc.	Mark
1	5,728	320	0,040	39	0,103	0,040	M
2	6,502	791237	99,960	37744	99,897	99,960	
Total		791557	100,000	37783	100,000		

Condições de Análise
Coluna RP-C18, 4,6x250mm - Fluxo: 1mL/min
Solventes: A=TFA 0,1%/H2O, B=CH3CN/H2O 9:1 com TFA 0,1%
Detector: 220nm - Gradiente: 5 a 100% de B em 25 min.

Peptide 4: NGDRGETGPAGPAGPAGPAGAR

AMINOTECH PESQUISA E DESENVOLVIMENTO - ANÁLISE POR HPLC

C:\LabSolutions\Data\Project1\23115-LCF01.lcd

Acquired by	: System Administrator
Sample Name	: 23115-LCF
Sample ID	: 23115-LCF
Tray#	: 1
Vial#	: 1
Injection Volume	: 20
Data File	: C:\LabSolutions\Data\Project1\23115-LCF01.lcd
Method File	: C:\LabSolutions\Data\Project1\AmT-AnalCOLETA-ISO.lcm
Report Format File	: C:\LabSolutions\System\DEFAULT.lsr
Month-Day Acquired	: 14/11/2023
Month-Day Processed	: 14/11/2023

H2-Asn-Gly-Asp-Arg-Gly-Glu-Thr-Gly-Pro-Ala-Gly-Pro-Ala-Gly- Pro-Ala-Gly-Pro-Ala-Gly-Ala-Arg-COO

C:\LabSolutions\Data\Project1\23115-LCF01.lcd

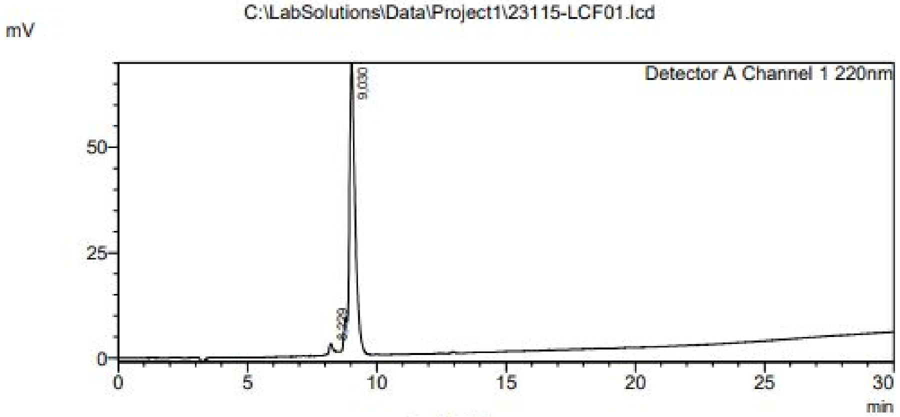

Peak Table

Detector A Channel 1 220nm

Peak#	Ret. Time	Area	Area%	Height	Height%	Conc.	Mark
1	8,229	21501	2,398	2295	3,506	2,398	
2	9,030	875264	97,602	63165	96,494	97,602	
Total		896765	100,000	65459	100,000		

Condições de Análise
Coluna RP-C18, 4,6x250mm - Fluxo: 1mL/min
Solventes: A=TFA 0,1%/H2O, B=CH3CN/H2O 9:1 com TFA 0,1%
Detector: 220nm - Gradiente: 5 a 100% de B em 25 min.
